# Exploring Determinants of Condom Use among University Students in Sudan

**DOI:** 10.1007/s10508-019-01564-2

**Published:** 2020-02-13

**Authors:** Husameddin Farouk Elshiekh, Ciska Hoving, Hein de Vries

**Affiliations:** Department of Health Promotion, CAPHRI School for Public Health and Primary Care, Maastricht University, P.O. Box 616, 6200 MD Maastricht, The Netherlands

**Keywords:** Sudan, HIV, Condom use, University students, I-Change Model

## Abstract

Increasing numbers of university students in Sudan are at risk of contracting HIV because of their engagement in condomless sex. A comprehensive and culturally accepted condom promotion program could potentially reduce this threat substantially. However, little is known about the sociocognitive determinants of condom use in this population: information that is required to develop such HIV prevention programs. Therefore, in August 2014, we conducted 30 semi-structured individual interviews with male and female students (both currently sexually active and nonactive) to explore determinants of condom use based on the I-Change model. Data were analyzed using Nvivo 10. The results suggest that barriers to condom use among university students include misconceptions about condom use, negative attitudes toward condom use, lack of social support, low self-efficacy to use condoms, and poor action planning. Sexual health promotion should, therefore, address these aspects to successfully promote condom use among sexually active students and subsequently reduce the risk of HIV transmission.

## Introduction

Since 2001, the annual number of new HIV cases and AIDS-related deaths has declined globally. However, many countries in the Middle East and North Africa (MENA) region are still suffering from the rising trends in new HIV infections due to slow response to the HIV epidemic and reluctance to address culturally sensitive issues, such as sex practices before marriage (Gökengin, Doroudi, Tohme, Collins, & Madani, 2016).

Sudan is one of the largest African countries in the MENA region. Following the separation of South Sudan in 2011, the total population in Sudan has become around 34 million: mostly Islamic with an almost equal gender distribution. Adolescents and young adults (10–35 years) represent almost half of the population. It is estimated that about 46% of the population lives below the poverty line. In addition, having military conflicts, substantial subsequent population movement and being bordered by countries with high HIV prevalence put the Sudanese population at increased risk of HIV infection (Sudan National AIDS Program [SNAP], 2014).

Like in the other Islamic countries in the MENA region, male circumcision is universal and abstinence from sex until marriage is a religious obligation. These facts had previously contributed to preventing HIV spread in Sudan. However, more young people in Sudan become involved in premarital sex because of the delayed marriage due to poverty. A study among female sex workers (FSW) in Sudan has also shown that most of them entered sex work to support their dependent family members and only few of them had jobs other than sex. Of them, 71% were married, 51% were married before the age of 18 and only 36% of them reported using condoms consistently (Abdelrahim, 2010). Intimate partner violence (IPV) as an important risk factor for HIV is recognized as a growing problem in the MENA region (Dworkin, Kambou, Sutherland, Moalla, & Kapoor, 2009). However, no official national data about its prevalence in Sudan are available. Coverage with HIV care services is still very low in Sudan. It has been estimated that less than 40% of people living with HIV/AIDS know their status and less than 15% of them are on treatment (UNAIDS, 2018).

In 2016, it was estimated that 56,000 [34,000–87,000] people were living with HIV in Sudan and the estimated number of new HIV infections in the same year was 5000 [1900–9400]. HIV prevalence among sex workers (FSW) and men practicing sex with men (MSM) was 1.3% and 1.4%, respectively (UNAIDS, 2017). The most commonly reported mode of HIV transmission in Sudan was unsafe heterosexual sex practices (Gökengin et al., 2016), such as having sex without using a condom.

Previous surveys conducted in 2002 by the SNAP revealed that HIV prevalence among university students was 1.1% (SNAP, 2002). Furthermore, sexual activity among university students in Sudan increased from 6.5 to 12.5% between 2002 and 2010 (SNAP, 2002, 2010). However, condom use as a primary preventive measure against HIV infection is still very low among this population. In 2010, only 20% and 32% of the sampled university students reported using condoms during their first-ever and latest sexual intercourse, respectively (SNAP, 2010).

As sexual practices are considered sensitive issues that are not openly discussed in the conservative community of Sudan (Mohamed & Mahfouz, 2013), little is known about the individual determinants of condom use among university students within this community. A recent study identified knowledge about AIDS transmission, education, type of sexual partners and experiencing condom problems such as tear as main predictors of condom use among visitors to voluntary counseling and testing (VCT) centers in Khartoum (Mohamed, 2014). However, visitors to VCT centers may not represent other university students. Moreover, the study only addressed a limited number of, mostly distal, sociocognitive determinants of condom use (e.g., knowledge). Recently, condom promotion interventions based on behavioral change theories have succeeded in promoting condom use in similar African communities (Mmbaga et al., 2017). This implies that further identification and understanding of the sociocognitive determinants of condom use are needed by public health workers to develop health communication materials for sexually active students or those at risk of becoming sexually active about condom use and HIV prevention. Therefore, this study aimed to explore the sociocognitive determinants of condom use among students, both currently sexually active and abstainers.

**Fig. 1:**
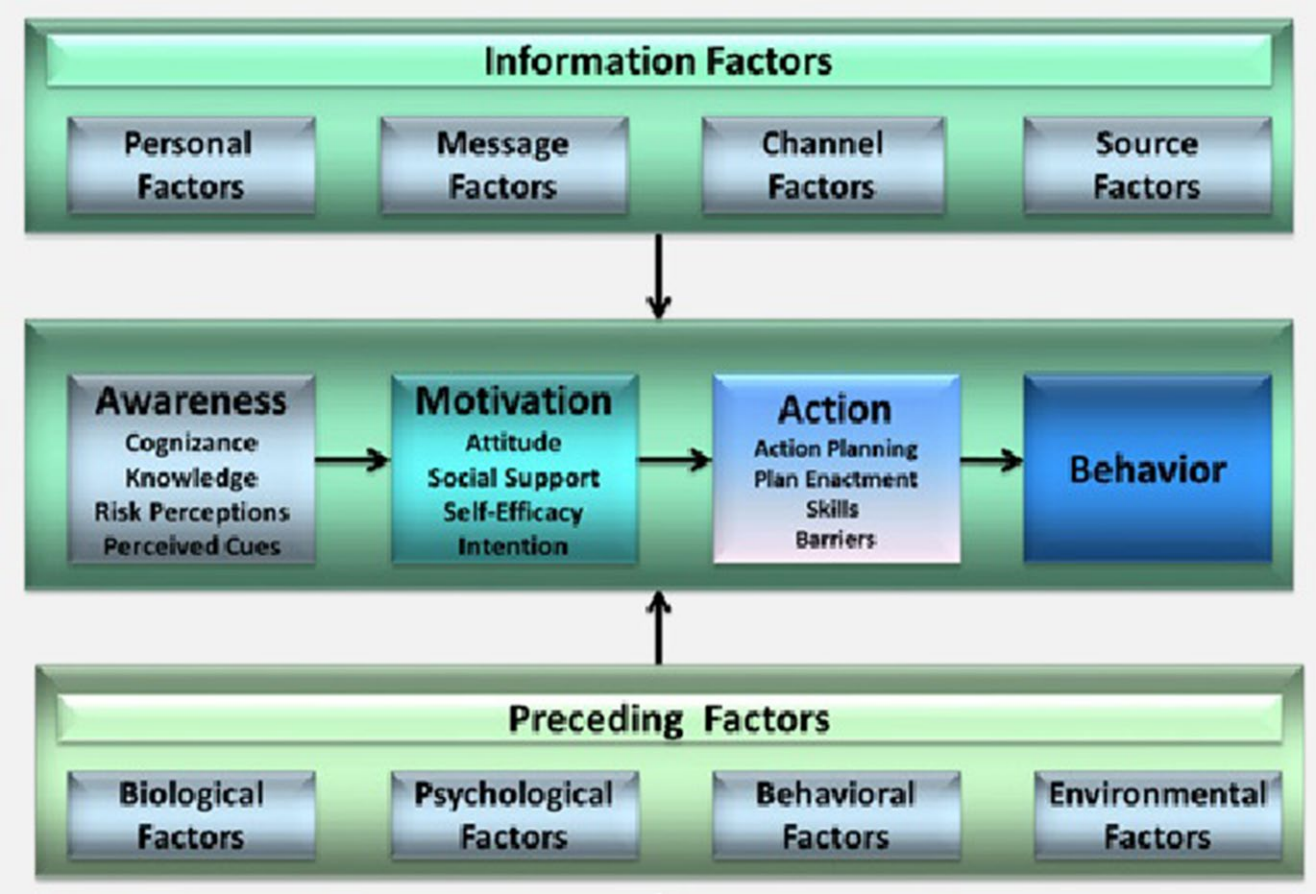
Integrated behavioral change (I-Change) model

To investigate the impact of sociocognitive determinants on condom use, we used the Integrated Model for Change (the I-Change Model, see Fig. 1) as a theoretical framework. This model integrates several social cognitive theories such as the Theory of Planned Behavior, Bandura’s Social Cognitive and Prochaska’s Transtheoretical Model, the Health Belief Model and Implementation, and Goal Setting Theories (Broekhuizen, van Poppel, Koppes, Brug, & van Mechelen, 2010) and has been successful in predicting health behaviors, including sexual health behaviors (de Vries, 2017; de Vries et al., 2014; de Vries, Mesters, Steeg, & Honing, 2005; Dlamini et al., 2009; Eggers et al., 2016; Huver, Engels, & de Vries, 2006). According to the I-Change Model, the behavioral change process has three phases: awareness, motivation and action. Each of these three phases has its relevant determinants. In relation to this study, the model assumes that condom use pre-motivational awareness phase is determined by a person’s cognizance of his/her sexual behavior, accurate knowledge about HIV and condom use and a person’s risk perceptions of how serious HIV is and how likely it is to get HIV if practiced condomless sex. This phase is also determined by the cues that prompt a person to use condoms consistently such as the death of a relative with AIDS. According to the I-Change Model, the motivational phase has three determinants: attitude, social influence and self-efficacy. In relation to this study, a person’s attitude toward condom use is his or her perception of the cognitive and emotional advantages and disadvantages of using condoms consistently. The social influence on a person’s behavior includes social support, norms and modeling. In relation to this study, social norm concerns an individual’s perception of what others in his community believe about condom use, social modeling is the individual perception of condom use behavior among the community members, and social support concerns the support in favor of healthy sexual behavior received from others. Self-efficacy is defined as the person’s perception of his capability to use condoms consistently and how difficult a person regards realizing the desired healthy behavior. The post-motivational action phase consists of action plans such as the plans required to prepare oneself and initiate condom use and the coping plans needed to overcome barriers. This phase is also determined by a person’s self-efficacy, skills and barriers. As assumed by the I-Change Model, these motivational processes are determined by predisposing biological (e.g., sex), psychological (e.g., personality), social (e.g., condoms availability) and information factors (e.g., messages quality and channels) (de Vries et al., 2005).

## Method

### Participants

To obtain an in-depth understanding of students’ condom use and the sociocognitive determinants of this practice, we conducted semi-structured individual interviews (Nshindano & Maharaj, 2008). Due to the sensitive nature of the study topic and the likelihood of refusal to participate due to religious or social reasons, focus groups were deemed unsuitable.

Thirty male and female university students from six different universities were interviewed in this study. Initially, a purposive heterogeneous sample of 15 university students was invited by the HIV counselors working at these universities to participate in the study. Because of the sensitivity of talking about sexual behaviors among this population, those who agreed to be interviewed were asked whether they would know other students that would be willing to participate in the study. Students thus approached other students and invited them to be interviewed (snowball recruitment). Interviews continued until saturation was reached (Kitzinger, 1995).

### Measures and Procedure

The I-Change Model served as the theoretical framework for the study. Therefore, a deductive approach was used to guide the development of the semi-structured interview guide (Elo & Kyngäs, 2008). The interview guide was designed to facilitate the exploration of condom use behavior and its pre-motivational, motivational and post-motivational determinants, including knowledge, risk perception, cues to action, attitude, social influence, self-efficacy and action plans. To put the respondents at ease and build rapport, the interviews started with the easy guide questions, and then the interviewers probed and encouraged the participants to talk about the more sensitive issues around their sexual behavior (Gill, Stewart, Treasure, & Chadwick, 2008). Table 1 summarizes the interview guide that was used during interviews. A pilot was undertaken with five university students other than those who participated in the study, and according to its results, minor linguistic changes were made in the interview guide.

**Table 1:** Summary of the study interview guide

A. General personal and demographic data	Age	
	Gender	
	Marital status	
	Type of university	
	Field of study	
	Family residence	
	Current condom use practices	
B. Exploring participant’s behaviors and beliefs about condom use	Knowledge	B.1. What do you know about condoms and consistent condom use in general?
	Risk perception	B.2. What are/could be the risks if you practice sex without condoms?
	Attitude	B.3.1. What are/could be for you the advantages of using condoms during sexual practices?
		B.3.2. What are/could be for you the disadvantages of using condoms during sexual practices?
		B.3.3. What role—if any—could consistent condom use play to protect you from getting HIV and other STIs?
	Social influence	B.4.1. Who would support you to use condoms during sexual practices?
		B.4.2. Who would be against you using condoms during sexual practices?
	Self-efficacy	B.5. When will it be difficult for you to use condoms during sexual practices?
	Other factors	B.6.1. What are the other factors which encourage you to use condoms during sexual practices?
		B.6.2. What are the other factors which prevent you from using condoms during sexual practices?

The individual interviews were conducted in August 2014 by either the principal researcher or one of two well-trained HIV counselors (a male and a female counselor). Most of the interviews (*n* = 21) were conducted inside the universities in private counseling rooms at the voluntary counseling and testing (VCT) centers. The remaining interviews took place at the students’ current residence (i.e., hostels). Each interview lasted between 50 and 75 min. The interviews were audio-recorded following written informed consent from each participant. However, most female participants refused to record their interviews, and detailed written notes were taken for those interviews instead. The interviews were conducted in Arabic, and all the audio-recorded interviews were transcribed verbatim before starting data analysis.

### Data Analysis

Initially, the transcribed interview data and written notes were revised by the research team. Based on the interview questions and the transcribed data, an initial coding scheme was developed by the principal researcher and the research assistants. Next, the transcripts were coded using Nvivo version 10. Additional subcodes were added to the coding scheme during the coding procedure. To check the validity of the coding process, two transcripts were fully coded by the principal researcher and one of the research assistants. Then, all transcripts were coded for a selection of codes (*n* = 5) by both of them (double coding). The results showed agreement percentage of 98.6 (94.3–100) and kappa of 0.81(0.28–1), which indicates good inter-coder reliability. Important themes identified were supported by quotes from the participants’ interviews.

### Ethical Consideration

Informed consent was obtained from all respondents included in the study before participation. Students were informed that their participation was voluntary and their confidentiality was assured throughout the interviews. To maintain the privacy and confidentiality of the participants, only the gender and age of the participants were linked to their quotes. Other identifiers such as their names, address or universities were removed during verbal transcription of the recorded interviews. In addition, the recorded interviews will be destroyed 5 years after the completion of the study.

## Results

### Description of the Sample

The sample included 16 male and 14 female students studying at six different universities in Khartoum State (Sudan). They varied in their sexual histories, socioeconomic statuses, backgrounds (rural or urban), fields of study, current academic year, and the type of their universities. Their age ranged from 18 to 24 years (M age, 19). Eleven reported being currently sexually active (six males and five females) (Table 2).

**Table 2:** Characteristics of study participants

Characteristic	Number	Percentage
*Gender*		
Male	16	53
Female	14	47
*Age*		
18–20 years	13	43
21–24 years	17	57
*Family residence*		
Khartoum State	20	67
Other states	10	33
*Type of university*		
Public	12	40
Private	18	60
*Field of study*		
Medical faculty	15	50
Education and arts	15	50
*Sexual activity*		
Sexually active	11	37
Abstainers	19	63

### Condom Use among University Students

The majority of the sexually active male and female participants reported that most of their sexual practices were unprotected. Only a few of them were using condoms consistently. Compared to the students who had never attended HIV educational programs, those who did stated that they were more likely to use condoms consistently.

I have attended training about HIV held by an organisation in which we were told that condom use prevents HIV. I became more convinced when I started using condoms consistently (21-year-old male, sexually active).

### Pre-motivational Determinants

#### Knowledge and Misconceptions About HIV and Condom Use

To explore their knowledge about HIV and condom use, the participants were asked what they knew about HIV and its transmission and prevention. They were also asked what they knew about condoms and whether they knew how to use condoms correctly. No knowledge difference was observed between male and female students. All of the participants knew that HIV could be transmitted sexually, and more than half of them were aware that condom use could prevent both HIV transmission and acquisition. However, the majority had the only superficial knowledge, if any, about how to use condoms and only those participants who attended HIV education programs seemed to know how to use condoms correctly. The majority of the sexually active participants gained detailed knowledge about condom use several years after they had started practising sex.

The first time I heard about the condom was three years ago. I started practising sex many years before that, but I did not have enough knowledge about using condoms (24-year-old male, sexually active).

Peers were the main source of knowledge about condom use among both male and female participants.

Misconceptions about condoms and their use were prevalent among male and female students, including both the sexually active and abstainers. Few participants questioned the protective role of condoms.

The first time I heard about the condom was during my secondary school study. I used to believe that it prevents HIV transmission and I used to argue with my friends who do not use it. Now, I believe that condoms only prevent pregnancy and have no other advantage. It is quite possible to get HIV even if you use condoms. I came to this conclusion after discussions with my peers and logical thinking (21-year-old male, sexually active).

Condoms protect only men against sexual diseases (21-year-old female, sexually active).

A few male participants also had the misconception that condom use could be associated with physical harm to both sex partners.

When you ejaculate, the penis will not have a space to ejaculate the semen since the condom will block the opening. Semen will remain inside the penis and cause harm. The condom also affects the muscles of the penis, and it may slip during sex and remain inside the vagina and cause health problems (18-year-old male, abstainer).

#### Risk Perception

The participants were initially asked about any risks they will be exposed to if they practiced sex without condoms. To explore their perception of susceptibility to HIV, they were asked how likely they believed they would contract HIV if they practiced condomless sex. They were also asked how serious HIV is to explore their perception of severity. Most of the male and female participants perceived the high risk of getting HIV if they practiced condomless sex. Almost all of them also indicated that HIV is a serious disease that not only kills but also destroys the social life of infected people.

These practices are associated with the risk of getting AIDS. We, as university students, are more exposed to this risk because these practices are more common in our university community and some students may be infected (18-year-old female, abstainer).

Despite this, the majority of the sexually active participants still practiced condomless sex, especially during spontaneous practices.

One day I met a girl whom I know well that she practises sex with many people and might be infected. She was so lovely, and I was so excited that I had sex with her without a condom (23-year-old male, sexually active).

In addition to the risk of getting HIV, the majority of the participants were also concerned about the risk of getting pregnant. Females and condom users reported higher pregnancy risk perception than males and nonusers. They also perceived the serious social consequences of illegal pregnancy.

I always remember pregnancy and its social and legal consequences. It is better for me to spend money to buy condoms to avoid pregnancy (21-year-old male, sexually active).

#### Cues to Action

When asked about the cues that encouraged them to use condoms consistently, consistent condom users reported different cues. About half of them mentioned having previous experience with people living with HIV/AIDS.

The first time I saw an HIV patient was in a health education activity. He looked very ill, and I was scared when I saw him. Since that time, I have become more careful and never practised sex without a condom (24-year-old male, sexually active).

A few participants also explained how having easy access to condoms encouraged them to use condoms consistently.

The main thing that encourages me to use condoms is that it is always easy for me to get condoms from the pharmacy where my close friend is working (19-year-old male, sexually active).

### Motivational Determinants

#### Attitude Toward Condom Use

Participants’ attitudes toward condom use were explored by asking them what they believed concerning the advantages and disadvantages of using condoms. The students’ attitudes toward condom use were quite variable. The majority of the sexually active participants who were consistent condom users had a positive attitude toward condom use. Most of the male students focused on the role of condoms in the protection against HIV and other sexually transmitted diseases. For most of the female students and a few male students, the most crucial advantage was preventing unwanted pregnancy.

It alleviates my fears of becoming pregnant. (18-year-old female, sexually active).

When I use condoms, I can get rid of the fears of having illegal pregnancies, especially if I know that the girl has relations with other boys. I may be accused of a pregnancy caused by someone else (if I practice sex without condoms) (23-year-old male, sexually active).

A few male participants also affirmed that using condoms helped them to engage in new sexual relations with those girls who preferred protected sex.

It helps me to gain new relations with girls. Those girls who are keen to have sex only with those who always use condoms will prefer me to other students (who are not using condoms) (21-year-old male, sexually active).

On the other hand, the majority of the sexually active students who were not consistent condom users expressed a negative attitude toward condom use. Both male and female participants perceived some common physical and emotional disadvantages of condom use. However, it was observed that most of the male students were more concerned about the perceived physical disadvantages of condom use.

I used to hear that condoms negatively affect sexual pleasure and decrease the size of the penis. My friends also told me that using condoms for a long time could affect masculinity and weaken ejaculation. They can also cause ulcers in the penis (24-year-old male, sexually active).

In contrast, the majority of the female participants seemed to be more concerned about the emotional disadvantages of using condoms. They believed that condom use could be interpreted as a lack of trust in the partner, hence affecting their emotional relationship. They also attributed condom use to causing discomfort and reducing sexual satisfaction.

Some of my sexual partners feel that condoms minimise sexual pleasure. Therefore, I feel discomfort with it (19-year-old female, sexually active).

In addition to this, a minority of the participants believed that condoms are costly and their use could provide a false sense of protection if misused.

Almost all of the male and female abstainers expressed a negative attitude toward condom use and talked about several religious, social and moral disadvantages. As they believed, condom promotion and distribution would justify and encourage sexual practices, destroy morality and spoil the youth. They considered premarital sexual practices more harmful than HIV itself.

They claim that condoms prevent AIDS. The problem is that even if we stop AIDS by using condoms, we will destroy the morals of young people. There is no benefit in preventing the spread of AIDS if this causes the spread of another disease; the moral decay and disintegration of families (20-year-old male, abstainer).

#### Social Influence on Condom Use

To study social influences on condom use, social norms, social pressure and support, and social modeling were explored.

#### Social Norms

The participants were asked about the prevailing norms associated with sexual practices and condom use and how these norms influenced their condom use behavior. In Sudan, the prevailing social norms prohibit the open discussion of sex and sex-related issues. All of the participants agreed that condom use was rarely discussed in public media. As expressed by most of the participants, virginity at the time of marriage remains a virtue in Sudan and any type of sex outside marriage is considered sinful. Females’ extramarital sex was believed to be associated with a social stigma and a negative impact on their family reputations.

Everybody will criticize me if they know that I have sexual relations. I am not the only one who will be affected but also the whole of my family. Even in the future, anyone will hesitate to become engaged with me or even with anyone of my sisters if he knows that I used to have sexual relations (21-year-old female, sexually active).

In addition, all of the participants talked about religion and religiosity and believed that religious principles act against premarital sexual behaviors.

These practices take me away from Allah because they are not allowed in Islam. Allah will not be satisfied with us. When I avoid sexual practices, I will maintain my dignity and preserve my family`s reputation (24-year-old female, abstainer).

Because of these social norms and religious values prohibiting sex outside marriage, both sexually active male and female students experienced difficulty in getting condoms. Both of them also declared that they could not use condoms consistently because keeping condoms with them would indicate their intention to practice sex. Female students were also affected by the prevailing social norm prohibiting their negotiation about sex and condom use.

It is the norm. If I ask for the condom, my partners will refuse just because they are men (23-year-old female, sexually active).

According to the prevailing concept of “natural love,” practicing sex without condoms was viewed as evidence of faithfulness to the partner.

Sometimes, I feel that it is more trusting to practise sex with my partner naturally and without a barrier (21-year-old male, sexually active).

#### Social Support and Pressure

Social support and pressure was explored by asking the participants who supported them to use condoms during sexual practices and who discouraged them. Regarding the influence of parents and other family members on condom use, almost all of the students agreed that parents and other family members support and advocate only abstinence and never discuss using condoms during sexual practices.

I cannot talk with my dad or mom about sex because they are not as close to me as my friends (18-year-old male, sexually active).

Politicians and policymakers were believed to be against condom use and resist condom distributions programs at universities. One of the participants pointed to the argument about condom promotion and distribution in the parliament of one of the provinces of Sudan saying:

Condom distribution among university students was discussed in the parliament of the Eastern Province one month ago. One of the members strongly resisted this and criticised the minister of health who suggested condom distribution as a solution to the problem of HIV among university students. People believe that distributing condoms will legalise its use [in extramarital sexual practices] and will send an encouraging message. The minister then denied his statement as I read in the newspaper (20-year-old male, abstainer).

Regarding the influence of religious leaders, all of the participants agreed that religious scholars were the main opponents of condom use and condom distribution because they believed that this would encourage students to practice extramarital sex.

Our imam says that calling the youth to use condoms will spoil them and destroy the community and nothing will be gained from that (24-year-old male, abstainer).

HIV counselors and other healthcare workers were considered the main and sometimes only supporters of condom use. Many sexually active students described how they encouraged them to use condoms whenever they practice sex.

One day, I talked to a counsellor who asked me about my sexual behavior. When I told him that I intend to practice sex, he advised me not to do that before marriage. He also advised me to use condoms if I practice sex so as to avoid many risks such as AIDS (21-year-old male, sexually active).

Peers and sexual partners seemed to be very influential. Most of the participants stated that they were not able to discuss any sexual issues with anybody other than their friends and sexual partners.

I usually talk to my close friends because we are at a similar age. I tell them about many things that happen to me and listen to them. My peers are always persuasive (18-year-old male, sexually active).

Most of the consistent condom users indicated that they were encouraged to use condoms by their sexually active peers and sexual partners.

My friends and I are very close to each other. We share our secrets. Most of them use condoms consistently and keep condoms in their pockets so they may have a chance to practise sex at any time. They encourage me to use condoms, but nobody else talks to me about that (21-year-old male, sexually active).

On the other hand, those participants who did not use condoms explained that they were discouraged by his peers and sexual partners who never use condoms.

My friends always tell me that sex is more pleasurable without condoms. They call it natural love. Most of my sexual partners also discourage me from using condoms as they believe that it reduces pleasure (19-year-old male, sexually active).

#### Social Modeling

All of the female students who reported using condoms consistently and most of the male condom users believed that condom use was common among the students.

University students are well educated. Most of them use condoms consistently to prevent HIV and unwanted pregnancy (21-year-old female, sexually active).

In contrast, those who did not use condoms and the abstainers looked at condom use as a rare practice among university students.

Only a very small minority of them (university students) uses a condom during sex (20-year-old male, abstainer).

### Post-motivational Determinants

#### Self-Efficacy

To explore the students’ self-efficacy, they were asked about the difficult situations associated with condom use and their confidence in their ability to use condoms consistently even if they were confronted by impediments. It was observed that male and female students faced different challenges. The majority of the male students narrated how they failed to resist their lust when condoms were not available or refused by their sexual partner.

Although I was afraid of pregnancy, the first time I practised sex I did not use a condom because I did not have one at that time. It is my nature that when I come to do anything while I am excited; I fail to do it the proper way. We were in another family`s house, and I was afraid of being caught there (19-year-old male, sexually active).

One day, I arranged with a girl to have sex. I prepared everything and kept a condom in my pocket. When I came to put it on, she refused. I tried my best to persuade her but failed. She was so sexy that I could not resist my desires (23-year-old male, sexually active).

At the same time, most of the female students asserted that it was difficult for them to negotiate condom use with their intimate partners.

I sometimes find it difficult to resist having sex without condoms when my intimate partner refuses to use it because he will think that I do not trust him (18-year-old female, sexually active).

Concerning students’ self-efficacy and confidence, generally female students appeared to consistently have lower self-efficacy to use condoms than male students, especially when they needed to negotiate its use. Students who reported using condoms consistently were more confident in their ability to use condoms and persuade any sexual partner to use it.

I will never think of having sex without condoms as I know the danger of practising sex without it. I always put AIDS in front of my eyes. We were told in a workshop that AIDS patients might look healthy and since that time, I have never practised sex without condoms (24-year-old male, sexually active).

#### Action Plans

The participants were asked how they planned to overcome difficulties and barriers to consistent condom use such as obtaining condoms, resisting sexual desire when excited and confronting sexual partner’s refusal. The majority of both male and female participants indicated to lack any action or coping plans to overcome these barriers. Only few of them had planned to buy condoms from remote pharmacies or ask their married friends to buy condoms for them. Few female students also talked about keeping condoms hidden.

I am very keen to keep condoms hidden in my handbag all the time to use them whenever I practise sex (19-year-old female, sexually active).

Another participant described how he avoided condomless sex when he lacked condoms saying:

Sometimes when I do not have condoms, I start with foreplay, but finally I give my partner any reason to avoid full sex like telling her that I have an appointment with somebody (24-year-old male, sexually active).

## Discussion

HIV prevention and control in any community requires the implementation of a combination of different interventions such as HIV testing, condom use, male circumcision, behavioral risk reduction, treatment of sexually transmitted infections and the use of antiretroviral medications (Kurth, Celum, Baeten, Vermund, & Wasserheit, 2011). This study focused on condom use as one of the effective HIV prevention interventions and sought to explore the sociocognitive determinants of condom use among university students in Khartoum using the I-Change Model as a theoretical framework.

### Pre-motivational Determinants

Several knowledge gaps and misconceptions about condom use were identified, such as causing physical harm to the user, being ineffective in preventing HIV and only protecting men against HIV. A previous study among dental students in Khartoum also showed that only 50% of the participants believed that condom use could prevent HIV (Nasir, Åstrøm, David, & Ali, 2008). These misconceptions seemed to influence condom use among students. The observed lack of knowledge about how to use condoms properly is another significant finding as recent research among this population has shown that experiencing problems with condom use such as condom breakage, slippage and fit is a predictor of condom use (Mohamed, 2014).

Regarding risk perception, high level of perception of HIV severity was observed among both condom users and noncondom users. Additionally, no difference in perception of susceptibility to HIV was observed between the two groups or between male and female students. However, a study among university students in Zimbabwe highlighted the importance of differentiating between students’ personal risk perception and their perception of HIV risk of their fellow students (Nkomazana & Maharaj, 2014). Compared with the perceived health risk, the results also suggested that the perceived social risk of practicing premarital sex could be more influential on some students’ sexual behaviors. Perceiving the risk of pregnancy and its severe social, legal and religious consequences seemed to be associated with consistent condom use among the study population, especially female students. This association was also observed in some previous studies (Maharaj, 2006; Pleck, Sonenstein, & Ku, 1991). This indicates that the students’ perception of susceptibility and severity of unwanted pregnancy could be exploited to promote condom use for dual protection among this population. However, HIV prevention must be stressed as a distinct goal to avoid misinterpretation by those using other contraceptive methods (Steiner, Liddon, Swartzendruber, Pazol, & Sales, 2018).

The study suggested that having previous exposure to HIV-infected persons could have a role as an important cue for consistent condom use among both male and female students. This finding parallels a recent longitudinal study which has documented that knowing someone infected with AIDS or had died from it strongly predicted condom use among visitors to VCT centers in Khartoum (Mohamed, 2014). The death of a family member or knowing someone with AIDS also increases the perceived HIV severity, resulting in consistent condom use (Palekar, Pettifor, Behets, & MacPhail, 2008).

### Motivational Determinants

Regarding students’ attitudes toward condom use, both condom users and noncondom users believed that using condoms had the advantage of protecting them against HIV and preventing unwanted pregnancy. Consistent condom users also talked about feeling more relaxed during sex and expressed that using condoms would provide opportunities for having new sexual relations with condom-using sex partners. However, noncondom users perceived several disadvantages associated with condom use as well. Parallel to a previous study among college students (Randolph, Pinkerton, Bogart, Cecil, & Abramson, 2007), noncondom users believed that condoms reduce sexual pleasure. The perceived disadvantages also included being costly, affecting emotional relations and causing physical harm. The diverse nature of perceived advantages and disadvantages of condom use among this population indicates that to build positive attitudes toward condom use, students need to be encouraged to combine both cognitive and affective assessment of the behavior. Although the findings suggested that male and female students perceived different physical and emotional disadvantages of condom use, further research with a larger sample is required to explore this difference. This will help in developing more gender-sensitive and -specific messages to build positive attitudes toward condom use.

It was also observed that some sexually active participants were not consistent condom users despite believing in their role in preventing HIV and pregnancy. This observation may indicate that attitudes toward condoms are not the sole or most important determinants of condom use among this sample. This finding has also been observed in previous studies in other African settings showing that social norms and self-efficacy are also strong predictors of condom use (Eggers et al., 2016; Taffa, Klepp, Sundby, & Bjune, 2002).

Regarding social influence, several social norms seem to have a substantial negative impact on condom use among university students. These norms include the social norms prohibiting the open discussion about sex and sex-related issues. They also include the popular concept of natural sex among sexually active students and the gendered power relations preventing female students from negotiating condom use with their partners.

The strong influence of social norms in communities more anchored in family ties like Sudan has also been observed in some previous studies (Eggers et al., 2016; Guan et al., 2016; Sarkar, 2008). These social norms may have a significant influence as they interact with the other psychosocial determinants of behavior, resulting in low condom use among university students. For instance, sexual health education (SHE) is difficult to implement as it is considered against modesty and believed to promote premarital sex. This prevented the dissemination of knowledge about safe sex among those who are already sexually active. Lacking credible sources of knowledge opens the door for misconceptions to prevail, leading to low condom use.

Social norms, in addition to the religious values against extramarital sex, also influence students’ attitudes toward condom use. The participants who expressed negative attitudes toward condom use believed that condom use encourages the illegal extramarital sexual practices. Cultural norms affect students’ self-efficacy as well since many students find it difficult to buy or keep condoms for use. Although in some communities, carrying condoms indicates caring about oneself, it is perceived as a sign of intentions to commit a sin among this population. Despite all of these challenges, recent research among a similar population has suggested that using suitable language and observing the sexual norms of the community as a part of SHE can reduce resistance (Latifnejad Roudsari, Javadnoori, Hasanpour, Hazavehei, & Taghipour, 2013) and mitigate the negative impact of these social norms against condom use.

Islamic religious values prohibit all types of extramarital sex. Therefore, Muslim religious leaders in Sudan were viewed as the leading opponents of condom use. However, in some Muslim communities, like Uganda and Indonesia, religious leaders do participate in HIV prevention and advocate condom use, for those unable to abstain, through the teachings of Quran and Hadith calling for compassion and prevention of harm and disease (Hasnain, 2005). Similar to several previous studies (Bosompra, 2001; DiClemente, 1991; Zhang, Jemmott, & Heeren, 2017a), our study also identified peer influence as an important determinant of condom use among sexually active students. In addition to the effect of subjective peer norms, peers are the only social group among this population with whom sexual practices are discussed. Condom users were encouraged and supported by their condom-using peers and sexual partners who acted as role models and provided them with condoms. On the other hand, noncondom users were discouraged by those peers and sexual partners who had misconceptions and negative attitude toward condom use. Therefore, peers may be considered essential stakeholders in condom promotion programs among university students, and their involvement may facilitate the implementation and maximize the benefits of these programs.

On the other hand, and consistent with previous research (Mola et al., 2006; Zhang et al., 2017b), HIV counselors were believed to play a significant role in supporting and promoting condom use among university students. There are a few VCT centers located at some universities in Khartoum where HIV counselors can promote consistent condom use among the sexually active students visiting these centers through behavioral change counseling and condom provision.

### Post-motivational Determinants

Self-efficacy as an important motivational and post-motivational determinant of condom use was explored, and several situations that respondents perceived as difficult to use condoms were identified. The observed difference in self-efficacy levels between consistent condom users and nonusers suggested a possible association between condom use and self-efficacy among both male and female participants in this study. This association has been observed in previous quantitative studies as well (Baele, Dusseldorp, & Maes, 2001; Oppong Asante, Osafo, & Doku, 2016).

Among the perceived barriers to condom use, participants mentioned having sex with the intimate partner, partner refusal to use condoms, embarrassment associated with purchasing condoms, having no money to purchase condoms and not having condoms available when needed. Also in line with some previous studies (Nesoff, Kristin, & Delia, 2016), lacking the skills to negotiate condom use was a significant perceived barrier to condom use, especially among female students because of the prevailing social norms in Sudan prohibiting condom use negotiation by female partners. Recent research has also shown that women who practice sex for money are less likely to be able to negotiate condom use with their sexual partners. Therefore, it has been suggested that HIV prevention programs should also be structural (i.e., economic) to produce changes in the context in which condom negotiations take place (Dworkin et al., 2009).

Although a few participants described how they coped with barriers and difficult situations, most of them lacked coping plans to overcome condom use barriers. Having no coping plans seemed to be associated with lower condom use among this study population. A previous study suggests that to promote condom use, it is crucial to address these perceived barriers and enhance self-efficacy (Kaneko, 2007).

### Practice Implications

Considering the observed diversity of condom use determinants and barriers among this population, a comprehensive and culturally consistent behavioral change program is required. For such a program to be successful, it should be gender-specific in order to deal with observed differences between male and female students (Dworkin et al., 2009).

In addition to addressing misconceptions, risk perception and attitude, such a program should focus on peer influence and self-efficacy to promote condom use as they seem to be important determinants of condom use among university students.

Regarding peer influence on condom use behavior, the sexually active students could be provided with data about the percentage of the students who use condoms consistently, which is likely to be much higher than their expectations. Providing sexually active students with effective peer pressure resistance skills could also help them to resist peer pressure against condom use. Female students could be trained and encouraged to use assertive condom negotiation strategies such as direct request and withholding sex (French & Holland, 2013). To address the peer norms that look at practicing condomless sex as a sign of manhood and masculinity, male students should be convinced that consistent condom use is a healthy attribute of masculinity (Shai, Jewkes, Nduna, & Dunkle, 2012).

To enhance the student’s self-efficacy, different behavioral change methods could be incorporated such as verbal persuasion, reattribution training, self-monitoring and goal setting methods (Eldredge et al., 2016). These methods have been tested and found to be successful in changing behaviors and could be used to promote condom use. The verbal persuasion method relies on using messages suggesting that the participant possesses the capability to use condoms consistently. This method requires a credible source such as VCT counselors to convey these messages. In the reattribution training method, participants are helped to interpret previous failures to use condoms in terms of unstable attributions while previous successes are interpreted in terms of stable attributions. The self-monitoring method prompts the participants to keep a record of their condom use practices and encourages them to interpret and use the recorded data to find out when and why they fail to use condoms. Another useful method is goal setting where a participant makes a plan to assist him or her to become a consistent condom user. The participants must be committed to these goals in order to change their behavior (Eldredge et al., 2016). In addition to these cognitive skills, a previous study has suggested that focusing more on affect regulation skills can have a significant impact on condom use (Brown et al., 2012). Such skills include getting away from triggers for strong emotions either physically (situation modification) or cognitively (attentional deployment) (Houck et al., 2016).

Since our study looked only at the psychosocial determinants of condom use and did not take into account many other important factors such as poverty, violence and poor access to prevention services, future research also needs to pay attention to these factors and explore how they interact with these psychosocial determinants to influence students’ beliefs and sexual behaviors.

### Strengths and Limitations

This is the first qualitative study that deeply explores the determinants of condom use among both male and female university students in Sudan. Using the I-Change model as a theoretical framework facilitated the exploration of the important sociocognitive determinants of condom use. Furthermore, the lengthy interviews and the semi-structured interview guide with open-ended questions enabled the participants to talk freely about their behaviors and provide in-depth details. In addition, the sample included a heterogeneous group of students in terms of sexual histories, socioeconomic statuses, backgrounds (rural or urban), fields of study, current academic year and the type of their universities. However, the number of participants in the study was only 30 and some of them were recruited through snowball sampling, which means that this is not a representative sample and thus one cannot generalize the results to all university students in Sudan. Having both male and female and both sexually active and nonsexually active students in a sample of 30 participants challenged the thematic analysis and rendered it more difficult to identify subthemes because of the limited number of participants in each of these subgroups. Therefore, further research may be needed to assess the commonalities and differences in other regions and among the subgroups. Also no distinction was made in this study between male condoms, which are usually assumed, and female condoms. Therefore, a more explicit study on female condoms may be required. In addition, other relevant stakeholders were not included in the study, such as HIV counselors, parents, religious leaders and policymakers. Therefore, future research should involve these additional groups before program development.

### Conclusion

Promoting condom use is a big challenge in Sudan. Lack of knowledge, negative attitudes, lack of social support, low self-efficacy and poor action planning seem to be important factors in explaining the lack of condom use among this population. Therefore, a comprehensive behavior change program addressing all of these factors is urgently needed to promote condom use among sexually active university students. However, gender analysis of these factors with further quantitative studies is highly needed to develop gender-specific HIV prevention programs.
